# Metabolomics Comparison of Hanwoo (*Bos taurus coreanae*) Biofluids Using Proton Nuclear Magnetic Resonance Spectroscopy

**DOI:** 10.3390/metabo10080333

**Published:** 2020-08-14

**Authors:** Jun Sik Eom, Shin Ja Lee, Hyun Sang Kim, You Young Choi, Sang Ho Kim, Yoo Gyung Lee, Sung Sill Lee

**Affiliations:** 1 Division of Applied Life Science (BK21Plus), Gyeongsang National University, Gyeongsangnam-do, Jinju-si 52828, Korea; skyandstar07@naver.com (J.S.E.); 2437401@naver.com (H.S.K.); dudolboy301@naver.com (Y.Y.C.); 2 Institute of Agriculture and Life Science & University-Centered Labs, Gyeongsang National University, Gyeongsangnam-do, Jinju-si 52828, Korea; tlswk1000@hanmail.net; 3 Animal Nutrition and Physiology Team, National Institute of Animal Science, RDA, Jeonrabuk-do, Jeonju-si 55365, Korea; kim2051@korea.kr (S.H.K.); yoo3930@korea.kr (Y.G.L.)

**Keywords:** ^1^H-NMR spectroscopy, biofluids, *Bos taurus coreanae*, Hanwoo, metabolomic profiles

## Abstract

The aim of this study was to identify the metabolomic profiles of rumen fluid, serum, and urine from Hanwoo (*Bos taurus coreanae*), using proton nuclear magnetic resonance (^1^H-NMR) spectroscopy. In all, 189, 110, and 188 metabolites were identified in rumen fluid, serum, and urine, and 107, 49, and 99 were quantified, respectively. Organic acids, carbohydrates, and aliphatic acyclic compound metabolites were present at the highest concentrations in rumen fluid, serum, and urine, respectively. In addition, acetate, glucose, and urea were the most highly concentrated individual metabolites in rumen fluid, serum, and urine, respectively. In all, 77 metabolites were commonly identified, and 19 were quantified across three biofluids. Metabolic pathway analysis showed that the common quantified metabolites could provide relevant information about three main metabolic pathways, phenylalanine, tyrosine, and tryptophan biosynthesis; caffeine metabolism; and histidine metabolism. These results can be useful as reference values for future metabolomic research on Hanwoo biofluids in Korea.

## 1. Introduction

Metabolomics is a comprehensive approach that allows for the identification and quantification of all the metabolites in an organism which are affected by both genetic and environmental factors [1]. Since the first signal detection in 1945 by Bloch, Purcell, and their colleagues [2], nuclear magnetic resonance (NMR) spectroscopy has developed into the second most extensively used technique for metabolomic studies, after liquid chromatography mass spectrometry, and has been frequently applied in untargeted metabolic investigations. The concentration of each metabolite can also be identified and quantified from an NMR spectrum [1,2]. Metabolomic studies using NMR spectroscopy have been conducted to investigate human metabolic diseases [3], drug toxicity [4], and food quality discrimination [5]. Such studies using NMR-based have also been conducted in animals and plants [6].

Ruminants are mammals, such as cattle, goat, sheep, and deer, which are characterized by having complex stomachs designed to digest plant-based foods that are mainly made of cellulose and hemicellulose. These cellulosic components are decomposed into monosaccharides by several microorganisms present in the rumen (the first stomach), which occupies the largest volume to obtain energy amounts of approximately 70% to 80% [7]. Ruminants represent one of the most populous and economically important groups of animals in the world, as they are the source of meat, milk, leather, and other resources for humans [8]. The first metabolomics study using NMR spectroscopy was conducted in 1972 by Chandan et al. [9] and evaluated the components of milk, one of the biofluids of ruminants. Since then, similar studies of ruminant biofluids have been conducted [10]. Representative metabolomics data on various ruminant biofluids (rumen fluid, serum, plasma, milk, urine, and feces) have been applied to increase global productivity [11,12], identify metabolic changes due to heat stress [13,14], reduce methane emissions to prevent global warming [15,16], and explore biomarkers to aid on metabolic disease prevention [17,18,19].

Hanwoo (*Bos taurus coreanae*) is a native, taurine-type, small-sized cattle breed in Korea. In the past, Hanwoo were used extensively for farming, transportation, and religious sacrifice; however, over time, the breed has become a commercial source of meat [20]. Studies on Hanwoo reported that it provides meat of higher quality and improved taste [20]. Other studies have analyzed the volatile fatty acid (VFA) composition [21], growth performance, and blood characteristics of the breed [22]. In recent year, metabolomics research has been conducted by comparing the composition of Hanwoo steers rumen fluid with the VFA and monosaccharides metabolites, using proton NMR (^1^H-NMR) spectroscopy, high-performance liquid chromatography, and high-performance anion-exchange chromatography [23]. However, very few studies have been conducted on the metabolites that constitute Hanwoo biofluids, using ^1^H-NMR spectroscopy. In Korea, most existing metabolomics studies using NMR have focused on food [24], human biofluids [25], and monogastric animals [26].

The objective of the present study was to characterize the metabolomes of Hanwoo steers rumen fluid, serum, and urine using ^1^H-NMR spectroscopy. The collected data aim to serve as a reference guide for researchers who seek to apply a metabolomics approach in future Hanwoo research.

## 2. Results

### 2.1. Rumen Fluid Metabolites

**Figure 1:**
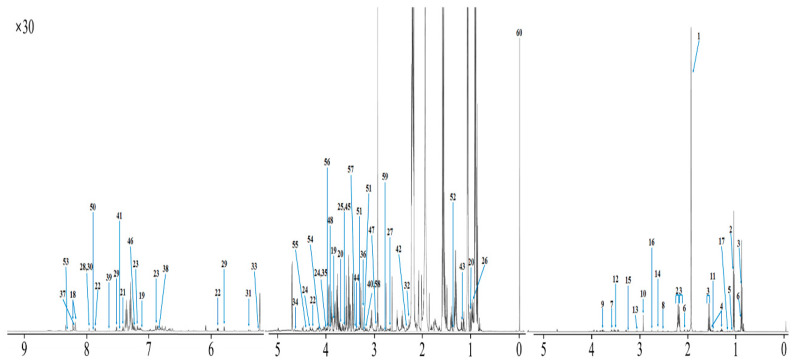
The representative spectrum ^1^H-NMR spectroscopy obtained from Hanwoo steers rumen fluid. Metabolite spectra numbers: (**1**) acetate, (**2**) propionate, (**3**) butyrate, (**4**) valerate, (**5**) isobutyrate, (**6**) isovalerate, (**7**) phenylacetate, (**8**) 3-phenylpropionate, (**9**) *N*-acetylglycine, (**10**) trimethylamine, (**11**) alanine, (**12**) 3-hydroxyphenylacetate, (**13**) cadaverine, (**14**) methylamine, (**15**) choline, (**16**) dimethylamine, (**17**) ethanol, (**18**) adenine, (**19**) anserine, (**20**) isoleucine, (**21**) phenylalanine, (**22**) uridine, (**23**) tyrosine, (**24**) galacarate, (**25**) threonine, (**26**) leucine, (**27**) methionine, (**28**) xanthine, (**29**) uracil, (**30**) ô-methylhistidine, (**31**) sucrose, (**32**) *p*-Cresol, (**33**) mannose, (**34**) *O*-acetylcholine, (**35**) creatine phosphate, (**36**) choline, (**37**) hypoxanthine, (**38**) gentisate, (**39**) thymidine, (**40**) malonate, (**41**) benzoate, (**42**) acetoacetate, (**43**) isopropanol, (**44**) 1,7-dimethylxanthine, (**45**) glycine, (**46**) thymol, (**47**) *N*,*N*-dimethylglycine, (**48**) *N*-nitrosodimethylamine, (**49**) creatine, (**50**) 4-pyridoxate, (**51**) O-phosphocholine, (**52**) 2-hydroxyisobutyrate, (**53**) histamine, (**54**) *N*-methylhydantoin, (**55**) tartrate, (**56**) betaine, (**57**) methanol, (**58**) dimethyl sulfone, (**59**) sarcosine, and (**60**) TSP. Detailed list of identified signals in this spectrum and corresponding metabolites in provided as Livestock Metabolites Database and Bovine Metabolites Database website.

The metabolites identified and quantified in the rumen fluid by using ^1^H-NMR spectroscopy are described in Figure 1, Supplementary Materials Figure S1a, and Supplementary Materials Tables S1–S4. In total, 189 metabolites were identified and categorized into 14 chemical classes. The classes with the most metabolites were others (31), carboxylic acids (28), and amino acids (22), while those with the highest concentrations were organic acids (44.88 mM), carbohydrates (1.605 mM), and amino acids (1.207 mM). In addition, 107 metabolites were quantified in the rumen fluid. The results in Figure 1 illustrate the identified and quantified compounds in rumen fluid. The top 30 concentrations of these metabolites quantified by ^1^H-NMR spectroscopy are described in Table 1. Among the metabolites quantified, acetate, propionate, and butyrate had the highest concentrations. In contrast, *N*-alpha-acetyllysine, 3-hydroxy-3-methylglutarate, and pyruvate had the lowest concentrations.

### 2.2. Serum Metabolites

**Figure 2:**
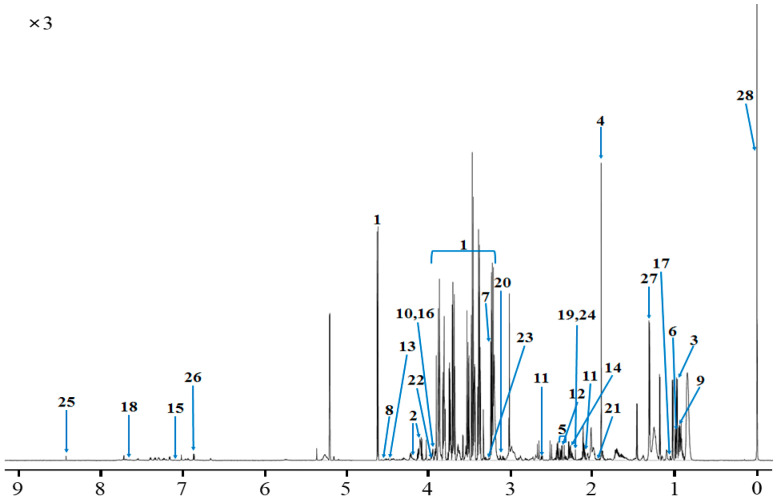
The representative spectrum ^1^H-NMR spectroscopy obtained from Hanwoo steers serum. Metabolite spectra numbers: (**1**) glucose, (**2**) ribose, (**3**) 2-hydroxyisovalerate, (**4**) acetate, (**5**) 3-hydroxybutyrate, (**6**) isoleucine, (**7**) betaine, (**8**) lactulose, (**9**) leucine, (**10**) creatine phosphate, (**11**) methionine (**12**) 3-hydroxyisovalerate, (**13**) ascorbate, (**14**) acetoacetate, (**15**) anserine, (**16**) creatine, (**17**) valine, (**18**) methylhistidine, (**19**) levulinate, (**20**) malonate, (**21**) *N*-acetyltyrosine, (**22**) clycolate, (**23**) trimethylamine *N*-oxide, (**24**) acetone, (**25**) formate, (**26**) tyrosine, (**27**) lactate, and (**28**) TSP. A detailed list of identified signals in this spectrum and corresponding metabolites is provided by the Livestock Metabolites Database and Bovine Metabolites Database website.

The metabolite identified and quantified in serum using ^1^H-NMR spectroscopy are described in Figure 2, Supplementary Materials Figure S1b, and Supplementary Materials Tables S1–S4. In total, 110 metabolites were identified and categorized into 12 chemical classes. The classes with the most metabolites were others (22), carboxylic acids (19), and carbohydrates (15) while with highest concentrations were carbohydrates (0.815 mM), organic acids (0.340 mM), and lipids (0.207 mM). In addition, 49 metabolites were quantified in the serum. The results in Figure 2 illustrate the identified and quantified compounds in serum. The top 30 concentrations of metabolites quantified by ^1^H-NMR spectroscopy are described in Table 2. Among the metabolites quantified, glucose, lactate, and 2-hydroxyisovalerate had the highest concentrations, whereas *N*-nitrosodimethylamine, *N*-acetylglucosamine, and 5-aminolevulinate had the lowest concentrations.

### 2.3. Urine Metabolites and Commonly Quantified Metabolites from the Three Biofluids

**Figure 3:**
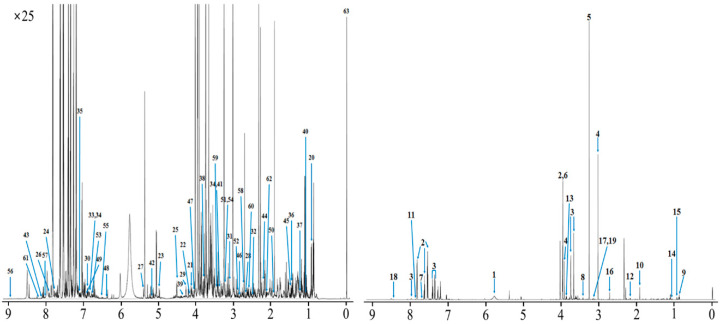
The representative spectrum ^1^H-NMR spectroscopy obtained from Hanwoo steers urine. Metabolite spectra numbers: (**1**) urea, (**2**) hippurate, (**3**) *N*-phenylacetylglycine, (**4**) creatine, (**5**) trimethylamine *N*-oxide, (**6**) glycolate, (**7**) 3-indoxylsulfate, (**8**) taurine, (**9**) 2-hydroxyvalerate, (**10**) acetate, (**11**) benzoate, (**12**) valerate, (**13**) 4-hydorxy-3-methoxymandelate, (**14**) 2-methylglutarate, (**15**) pantothenate, (**16**) dimethylamine, (**17**) malonate, (**18**) formate, (**19**) dimethyl sulfone, (**20**) 2-hydroxy-3-methylvalerate, (**21**) gluconate, (**22**) kynurenine, (**23**) mandelate, (**24**) salicylurate, (**25**) arabinose, (**26**) xanthine, (**27**) allantoin, (**28**) citrate, (**29**) galactarate, (**30**) xanthurenate, (**31**) cis-aconitate, (**32**) 3-phenylpropionate, (**33**) gentisate, (**34**) 4-hydroxyphenylacetate, (**35**) histamine, (**36**) 3-hydroxy-3-methylglutarate, (**37**) fucose, (**38**) guanidoacetate, (**39**) glucose-6-phosphate, (**40**) methylsuccinate, (**41**) 3-hydroxyphenylacetate, (**42**) xylose, (**43**) carnosine, (**44**) levulinate, (**45**) alanine, (**46**) succinylacetone, (**47**) creatinine, (**48**) urocanate, (**49**) vanillate, (**50**) *N*-acetyltyrosine, (**51**) carnitine, (**52**) *N*-methylhydantoin, (**53**) *p*-Cresol, (**54**) betaine, (**55**) 2,3,4-trihydroxybenzoate, (**56**) niacinamide, (**57**) theophylline, (**58**) sarcosine, (**59**) 1-3-dimethylurate, (**60**) methylamine, (**61**) oxypurinol, (**62**) hydroxyacetone, and (**63**) TSP. Detailed list of identified signals in this spectrum and corresponding metabolites in provided as Livestock Metabolites Database and Bovine Metabolites Database website.

The metabolite identified and quantified metabolites in urine by ^1^H-NMR spectroscopy are described in Figure 3, Supplementary Materials Figure S1c, and Supplementary Materials Tables S1–S4. In total, 188 metabolites were identified and categorized into 13 chemical classes. The classes with the most metabolites were others (39), carboxylic acids (27), and lipids (22) while those with the highest concentrations were aliphatic acylic compounds (52.20 mM), lipids (3.910 mM), and carbohydrates (1.953 mM). In addition, 99 metabolites were quantified in urine. The results in Figure 3 illustrate the identified and quantified compounds in urine. The top 30 concentrations of metabolites quantified using ^1^H-NMR spectroscopy are described in Table 3. Among the metabolites quantified, urea, hippurate, and *N*-phenylacetylglycine had the highest concentrations, whereas 3-pheylpropionate, salicylurate, and phenylacetate had the lowest concentrations. The results are summarized in Table 4, which reveals common metabolites across the three biofluids, including 19 quantified metabolites. Overall, carboxylic acid metabolites were the most quantified.

### 2.4. Statistical Analysis from Three Biofluids

To visualize the differences among the metabolites of the different three biofluids, we performed principal component analysis (PCA) and partial least square discriminant analysis (PLS-DA) (Supplementary Materials Figures S2 and S3). Both score plots revealed differences corresponding to rumen fluid, serum, and urine, which were well separated in PC1 (31.8%) and PC2 (21.8%) for PCA, and Component 1 (30%) and Component 2 (22.8%) for PLS-DA. These results highlight the differences in the classes and concentrations of the metabolites measured in rumen fluid, serum, and urine.

**Figure 4:**
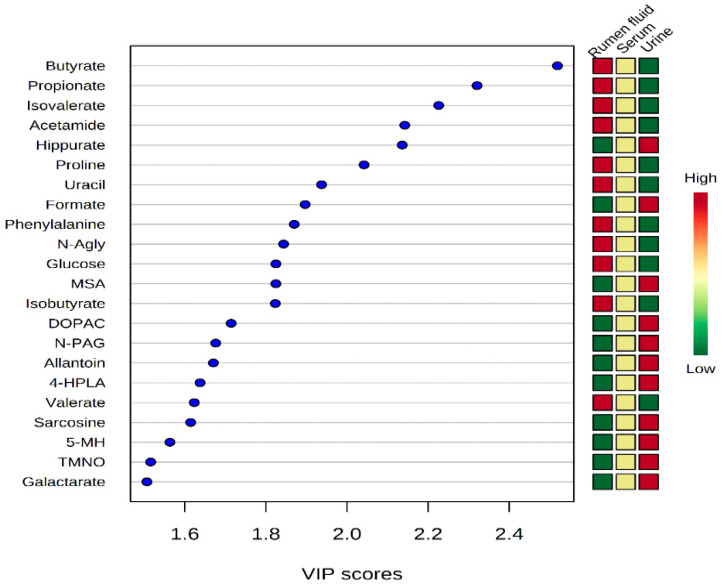
Variable importance in projection (VIP) scores of metabolites in rumen fluid, serum, and urine by ^1^H-NMR spectroscopy analysis. The selected metabolites were those with VIP > 1.5. Heat map with red, yellow, and green boxes on the right indicates high, medium, and low abundance ratio, respectively, of the corresponding metabolites in rumen fluid, serum, and urine. VIP score was based on the PLS-DA model. VIP score value: butyrate, 2.519; propionate, 2.3206; isovalerate, 2.2261; acetamide, 2.1425; hippurate, 2.1361; proline, 2.042; uracil, 1.9373; formate, 1.8968; phenylalanine, 1.8699; *N*-agly, 1.8437; glucose, 1.8247; MSA, 1.8246; isobutyrate, 1.8232; DOPAC, 1.7143; *N*-PAG, 1.6764; allantoin, 1.6705; 4-HPLA, 1.6377; valerate, 1.6234; sarcosine, 1.6145; 5-MH, 1.5633; TMNO, 1.5159; galactarate, 1.5065. Metabolites abbreviation: *N*-agly, *N*-acetylglycine; MSA, methylsuccinate; DOPAC, 3,4-dihydroxybenzeneacetate; *N*-PAG, *N*-phenylacetylglycine; 4-HPLA, 4-hydroxyphenyllactate; 5-MH, 5-methylhisidine; TMNO, trimethylamine *N*-oxide.

As shown in Figure 4, rumen fluid, serum, and urine presented completely different metabolomic profiles. Variable importance in projection (VIP) scores were used to identify which metabolites were responsible for the differentiation pattern in the PLS-DA plot. This analysis revealed that 22 metabolites were significantly different (VIP score > 1.5) between rumen fluid, serum, and urine (Figure 4). Moreover, in rumen fluid, 11 metabolites (butyrate, propionate, isovalerate, acetamide, proline, uracil, phenylacetate, *N*-acetylglycine, glucose, isobutyrate, and valerate) were significantly more concentrated than in serum and urine. In urine, 11 metabolites (hippurate, formate, methylsuccinate, 3,4-dihydroxybenzeneacetate, *N*-phenylacetylglycine, allantoin, 4-hydroxyphenyllactate, sarcosine, 5-methylhistidine, trimethylamine *N*-oxide, and galactarate) were also found at significantly higher concentrations than in rumen fluid and serum.

### 2.5. Metabolic Pathway Analysis

**Figure 5:**
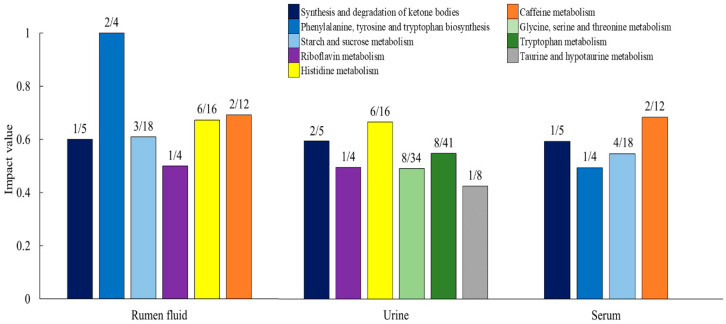
Metabolic pathways evidenced by enrichment analysis based on the metabolites common quantified in rumen fluid, serum, and urine (impact value ≥ 0.5). The *x*-axis represents the biofluid samples, and *y*-axis represents the impact value. The number on each bar graph is match status. Match status is hit/total. The total is the total number of compounds in the pathway; the hits is the actually matched number.

Metabolic pathway analysis for common quantified three biofluids was performed by using the MetaboAnalyst platform, as detailed in Figure 5. Overall, the phenylalanine, tyrosine, and tryptophan biosynthesis pathway showed the highest impact score, and six other pathways were described with an impact score higher than 0.5. These pathways included tryptophan metabolism, caffeine metabolism, histidine metabolism, riboflavin metabolism, starch and sucrose metabolism, and synthesis and degradation of ketone bodies.

## 3. Discussion

VFAs that can be found in the rumen, such as acetate, propionate, butyrate, valerate, isobutyrate, and isovalerate are the main sources of energy for ruminants [27]. Diets containing a high density of concentrates result in the absorption of more VFAs in the rumen [28], thereby in increased productivity by the ruminants. In contrast, as the rumen pH decreases, the risk of acidosis increases [29]. Previous studies by Ametaj et al. [17], Saleem et al. [30], and Wang et al. [31] reported that feed diets containing a high density of concentrates increased ethanol, ethanolamine, 3-hydroxybutyrate, dimethylamine, *N*-nitrosodimethylamine, glucose, propionate, butyrate, alanine, maltose, uracil, xanthine, phenylacetylglycine, phenylacetate, and biogenic amine (tyramine, putrescine, histamine, methylamine, and tryptamine) concentrations, whereas it reduced 1,3-dihydroxyacetone and 3-phenylpropionate concentrations. Our results include all metabolites associated with acidosis that were identified in previous studies, except for ethanolamine, phenylacetylglycine, tyramine, and putrescine. Hanwoo are fed a high density of concentrates until they are nearly 30 months of age. The primary reason for this type of diet is that it improves meat quality with high marbling, which is one of the main factors contributing for the high-quality grade of Hanwoo meat [32]. Therefore, the study of metabolites using Hanwoo rumen fluid could be used as an index to predict and prevent acidosis.

Ruminants produce approximately 80 million tons of methane (CH_4_), which accounts for about 33% greenhouse gas (GHG) emissions [33]. CH_4_ is a powerful GHG, with a global warming potential 28-fold higher than carbon dioxide (CO_2_) [34]. Given that CH_4_ is also associated with a 2% to 15% loss of dietary potential energy, productivity in ruminants is reduced while the rumen production of CH_4_ continues [33,35]. Ruminant CH_4_ is generated via two main methanogenesis routes in the rumen, both of which are carried out by archaea [36]. The hydrogenotrophic pathway converts hydrogen (H_2_) and CO_2_ produced by the bacteria, protozoa, and fungi to CH_4_ [37]. H_2_ and CO_2_ production is greatly influenced by numerous metabolites in the rumen. Saleem et al. [30] classified dimethylamine, methylamine, *N*-nitrosodimethylamine, formate, uracil, and threonine as metabolites related to CH_4_ emission in the rumen; among these, methylamine class metabolites are rapidly converted to CH_4_ through the methylotrophic CH_4_ emission pathway in the rumen [11]. *Methanosarcina barkeri* and *Methanosarcina mazeii* produce CH_4_ by using acetate, methylamine, and methanol, as well as H_2_ and CO_2_, which are present in the rumen [38]. The metabolites produced by the decomposition of choline by microorganisms in the rumen are known as acetate, ethanol, and ethylene glycol [39,40]. The methyl groups from choline are likely reduced to CH_4_ via a carrier, such as cobalamin or tetrahydrofolate, which are both abundant in methanogenic bacteria [41]. According to Asanuma et al. [42], in order to reduce CH_4_ emissions from the rumen, H_2_ used both in methanogenesis and (the formate must be reduced) as formate is converted into H_2_ or CO_2_ by formate dehydrogenase. Propionate and butyrate in the rumen are generated by using H_2_ [43,44,45] and dicarboxylic acid class metabolites like aspartate, malate, and fumarate are also generated using H_2_ [46,47]. According to Newbold et al. [48], fumarate, succinate, and acrylate must produce propionate, using H_2_ to reduce CH_4_ emissions due to the “4H_2_ + CO_2_ → CH_4_ + 2H_2_O” pathway in the rumen. Among them, the probability of using fumarate for H_2_ is 70%, and the probability of using acrylate for H_2_ is close to 100% [48]. When 2-oxoglutarate is converted to succinate, H_2_ is released and fumarate reuses these H_2_ molecules through succinate to produce propionate [48]. In addition, methionine in the rumen is produced by the methylmercapto group, using a methyl group as a CH_4_ emission substrate [41]. Therefore, high concentrations of acetate, methylamine, *N*-nitrosodimethylamine, dimethylamine, trimethylamine, trimethylamine *N*-oxide, choline, ethylene glycol, threonine, and uracil in the rumen are thought to increase CH_4_ emissions, whereas propionate, butyrate, malate, methionine, succinate, carnitine, and fumarate concentrations are thought to reduce CH_4_ emissions. Our results showed that CH_4_-related metabolites, except for malate, carnitine, and fumarate, were present in Hanwoo steers rumen fluid. Therefore, this work is expected to aid future research on CH_4_ reduction in Hanwoo.

Ruminants balance negative energy with relatively low-energy intake. Negative energy balance is associated with a higher risk of metabolic disorders [49], poor health, and infertility [50,51]. To compensate for the energy deficit, ruminants mobilize body reserves [51], such as body fat and muscle protein [52]. Weikard et al. [53] reported significant positive correlations between plasma carnitine and body weight. Carnitine plays a key role in cellular energy metabolism, mainly by transferring acyl groups from the cytoplasm to mitochondrial, thereby rendering the utilization of energy in feed and body stores more efficiently [53]. Glycine in plasma could be used as an indicator of energy balance and metabolic status in dairy cattle [54]. Glucose is not only used as an energy source but also as a precursor for synthesizing lactose in milk and is regulated by insulin [55]. Xu et al. [56] reported correlations between energy balance metabolites in the plasma and milk production, such as acetone, acetylcarnitine, aspartate, 3-hydroxyacetone, carnitine, creatinine, glycine, hydroxyproline, and thymidine. Our results also revealed that several metabolites associated with energy balance, including acetone, 3-hydroxyacetone, and creatinine, were present in the Hanwoo steers serum. As Hanwoo are not used for milk production, these results could be useful, as they are also directly related to body weight gain.

Bovine respiratory disease (BRD) is a multifactorial disease of notable welfare and economic significance to the global feedlot industry [57]. BRD is caused by association of physiological and environmental stressors prior to and upon feedlot admittance, for example, transportation, mixing of strange animals, and exposure to viral and microbial population agents [58]. According to Basoglu et al. [59], BRD increases 2-methylglutarate, phenylalanine, and phosphatidylcholine concentrations, while decreasing ethanol, dimethyl sulfone, propionate, acetate, allantoin, free cholesterol, and cholesterol concentrations. Blakebrough-Hall et al. [57] also found that phenylalanine, lactate, hydroxybutyrate, tyrosine, citrate, and leucine metabolites were of importance for distinguishing BRD-affected animals from healthy bovine. Our results showed that acetate, lactate, hydroxybutyrate, and leucine could be identified in the Hanwoo steers serum. BRD-related metabolites in Hanwoo were not the primary focus of our research; however, given that Hanwoo metabolites studies in serum are actively conducted, such work could be used as a reference.

Metritis is a uterine pathology that affects all uterine layers and causes decreased breeding rates, increased culling rates, increased veterinary costs, and decreased milk yield in dairy cows. Dervishi et al. [60] reported that cows with metritis harbor abnormal concentrations of metabolites associated with carbohydrate metabolism, acute phase proteins, and proinflammatory cytokines, which are first noticeable at an early phase (four and eight weeks) before parturition and the manifestation of clinical signs of metritis. Dervishi et al. [61] also reported in the urine of pre-metritic and metritic dairy cows can be detected excretion of monosaccharides and tricarboxylic acid cycle related metabolites, as well as amino acids and carbohydrates catabolites. Moreover, they suggested that, galactose, leucine, lysine, and pantothenate levels at eight weeks and a combination of histidine, isocitrate, lysine, O-phosphocholine, threonine, trans-aconitate, xylose, and 3-aminoisobutyrate at four weeks prior have predictive value and may function as potential risk biomarkers for cow susceptibility to metritis. Our results showed that the metabolites that were suggested by Dervishi et al. [61] as metritis biomarkers, including pantothenate, histidine, xylose, *O*-phosphocholine, and trans-aconitate, as well as amino acids, tricarboxylic acid cycle metabolites, monosaccharides, and carbohydrate catabolites, were all identified and quantified in Hanwoo steers urine. Therefore, this work may act as reference for future research on metritis in Hanwoo.

Lameness is a leg and hoof inflammatory condition that is associated with pain, resulting in impaired posture and gait of the animal [62]. The major negative effects of lameness are related to the postponed resumption of ovarian activity [63]. These symptoms could negatively affect ruminant productivity. Zhang et al. [64] reported that uracil, formate, *N*-*N*-dimethylglycine, and tyrosine were consistently lower, while lysine, pantothenate, hypoxanthine, and xylose were greater in the urine of pre-lame cows at eight and four weeks prepartum. Furthermore, during the week of lameness diagnosis, 2-hydroxyisobutyrate, 3-hydroxy-3-methylglutarte, 4-hydroxyphenylacetate, adipate, glycerate, tyrosine, and valine were less concentrated in the urine of lame-confirmed cows. Our results showed that the metabolites suggested by Zhang et al. [64], as associated with lameness, including formate, pantothenate, xylose, 2-hydroxyisobutyrate, 3-hydroxy-3-methylglutarte, 4-hydroxyphenylacetate, and glycerate metabolites, were identified in Hanwoo steers urine. Therefore, this work may also provide reference values for future research on lameness in Hanwoo.

## 4. Materials and Methods

All experimental protocols used in this study were approved by National Institute of Animal Science of Animal Nutrition and Physiology Team (Jeonju, Jeollabuk-do, Korea, NIAS20171082).

### 4.1. Animals and Collected Samples

Six Hanwoo steers (430 ± 21 kg) were included in this study. They were fed 1.2 kg of roughage and 3.0 kg of concentrate twice a day (09:00 and 18:00), with ad libitum access to mineral blocks and water. The dry matter (DM; #934.01) contents of the commercial roughage were 68.5 g/kg of crude protein (CP; #976.05), 10.5 g/kg of ether extract (#920.29), 91.2 g/kg of crude ash (CA; #942.05) [65], 590.7 g/kg of neutral detergent fiber (NDF), and 384.2 g/kg of acid detergent fiber (ADF) [66]. The contents for the commercial concentrates were 183.7 g/kg of CP, 27.8 g/kg of ether extract, 82.5 g/kg of CA, 216.5 g/kg of NDF, and 94.9 g/kg of ADF.

Rumen fluid, serum, and urine samples were collected before the morning feeding (09:00). Rumen fluid was collected from rumen calculated Hanwoo steers. The samples were centrifuged at 806× *g* and 4 °C for 15 min, to remove feed particles, and the supernatant was stored at −80 °C for later ^1^H-NMR spectroscopy analysis. Blood from the jugular neck vein was collected of each steer. The serum samples were centrifuged at 15,142× *g* and 4 °C for 5 min, and aliquots of the upper layer (serum) were stored at −80 °C for later ^1^H-NMR spectroscopy analysis. Urine samples were collected by hand-sweeping the perineum, thus stimulating each steer to urinate, and stored at −80 °C for later ^1^H-NMR spectroscopy analysis.

### 4.2. Sample Preparation for ^1^H-NMR Spectroscopy

Rumen fluid samples were centrifuged at 12,902× *g* and 4 °C for 15 min and collected at 300 μL of supernatant. Standard buffer solution (TSP; 2,2,3,3-d4-3-(Trimethylsilyl) propionic acid sodium salt) was added to 300 μL in deuterium oxide (D_2_O) solvent. The mixture solution (600 μL) was transferred to 5 mm NMR tube for ^1^H-NMR spectroscopy spectral analysis [8].

Saline buffer was prepared in NaCl concentration of 0.9% weight/volume in 100% D_2_O. The stored serum samples were centrifuged at 14,000× *g* and 4 °C for 10 min. The supernatant 200 and 400 μL of saline buffer was added to the 5 mm NMR tube for ^1^H-NMR spectral spectroscopy analysis [67].

Urine sample was utilized to 0.2 M sodium phosphate buffer (pH 7.0). The samples were centrifuged at 14,000× *g* at 4 °C for 10 min, and we collected 400 μL supernatant. Supernatant was added to 230 μL of buffer and was measured at pH 7.0 ± 0.1. The mixture solution (630 μL) was added to 2 mM TSP 60 μL, and the TSP concentration in the total solution was adjusted to 0.2 mM [68]. The prepared sample was transferred to a 5 mm NMR tube, for ^1^H-NMR spectroscopy spectral analysis.

The spectra of rumen fluid, serum, and urine samples were obtained on a SPE-800 MHz NMR–MS Spectrometer (Brunker BioSpin AG, Fällanden, Switzerland) at 298 K, using a 5 mm triple-resonance inverse cryoprobe with Z-gradients (Bruker BioSpin CO., Billerica, MA, USA). The pulse sequence used for the rumen fluid, serum, and urine were a Carr-Purcell-Meiboom-Gill pulse sequence collecting 64,000 data points with 128 transients, a spectral width of 16,025.641 Hz, a relaxion delay of 4.0 s, and an acquisition time of 2.0 s [69].

### 4.3. ^1^H-NMR Spectroscopy Data and Statistical Analysis

The processed spectra were imported the Chenomx NMR suite 8.4 software (Chenomx Inc., Edmonton, AB, Canada) for identification and quantification. The baseline and phase were matched for comparison between samples, using the NMR software. The following procedure was employed for qualitative and quantitative analysis of the metabolites in samples. The spectral width was 10 ppm and was referenced to the TSP signal at 0 ppm. The resources used were the Livestock Metabolite Database, Bovine Metabolite Database, and Chenomx library. Metabolite classification and quantitation were performed by using the Chenomx profiler program.

Statistical analyses of the metabolite data were conducted by using MetaboAnalyst version 4.0 [70], an open-source R-based program for metabolomics. The resulting metabolites were subjected to sample normalization by “sum”, data transformation by “log”, and data scaling by “pareto” during statistical analysis. Univariate Student’s *t*-tests were used to identify difference between metabolite profiles of the biofluid samples. Principal component analysis (PCA) and partial least square discriminant analysis (PLS-DA) were used as multivariate data analysis techniques, to generate a classification model and provide quantitative information for discriminating the metabolites. The different biofluid metabolites were determined on the basis of a statistically significant threshold of variable importance in projection (VIP) scores. Metabolites with VIP scores higher than 1.5 were obtained through PLS-DA.

Metabolic pathways analysis was performed using a *Bos taurus* pathway library. Metabolic pathways were common quantified, and different metabolites in biofluid metabolites of the other studied animals were statistically analyzed by MetaboAnalyst 4.0 for metabolic pathways analysis, which is based on database source by Kyoto Encyclopedia of Genes and Genomes [71].

## 5. Conclusions

^1^H-NMR spectroscopy and statistical analyses were employed to analyze the metabolites in Hanwoo steers rumen fluid, serum, and urine. The resulting metabolite measurements were mostly consistent with those reported in studies conducted outside Korea. This report will contribute for future Hanwoo metabolomic studies in Korea by serving as a reference guide.

## Figures and Tables

**Table 1:** Top 30 concentration (mean ± standard deviation) of metabolites quantified (*n* ≥ 4) in rumen fluid by ^1^H-NMR spectroscopy of Hanwoo steers.

Metabolites	Class	Concentration (μM/L)
Acetate	Organic acids	28,172.77 ± 4924.54
Propionate	Organic acids	8126.70 ± 1341.28
Butyrate	Organic acids	6021.97 ± 1140.22
Valerate	Organic acids	940.82 ± 187.60
Glucose	Carbohydrates	632.42 ± 387.16
Isobutyrate	Organic acids	495.55 ± 168.30
Isovalerate	Organic acids	470.08 ± 144.26
Acetamide	Organic acids	237.18 ± 47.79
Ribose	Carbohydrates	231.48 ± 50.74
3-phenylpropionate	Others	223.40 ± 55.69
Phenylacetate	Organic acids	220.22 ± 49.20
3-methylglutarate	Lipids	214.75 ± 67.05
Alanine	Amino acids	195.65 ± 51.88
Maltose	Carbohydrates	178.13 ± 278.73
Caprate	Lipids	160.80 ± 58.72
Proline	Amino acids	119.20 ± 40.69
*N*-acetylglucosamine	Carbohydrates	112.55 ± 16.91
Urea	Aliphatic acylic compounds	86.56 ± 55.54
*N*-carbamoylaspartate	Carboxylic acids	86.07 ± 33.83
Xanthine	Nucleosides, nucleotides	70.03 ± 12.87
*N*-acetylglycine	Carboxylic acids	61.42 ± 64.75
Glycine	Amino acids	61.26 ± 41.85
Uracil	Nucleosides, nucleotides	58.87 ± 17.19
Threonine	Amino acids	57.50 ± 12.14
Isoleucine	Amino acids	57.47 ± 22.42
Glycerate	Amino acids	52.57 ± 42.20
Lactulose	Carbohydrates	52.36 ± 40.70
Pyruvate	Carbohydrates	51.98 ± 27.61
3-hydroxy-3-methylglutarate	Lipids	51.50 ± 33.29
*N*-alpha-acetyllysine	Carboxylic acids	51.08 ± 53.04

**Table 2:** Top 30 concentration (mean ± standard deviation) of metabolites quantified (*n* ≥ 4) in serum by ^1^H-NMR spectroscopy of Hanwoo steers.

Metabolites	Class	Concentration (μM/L)
Glucose	Carbohydrates	603.60 ± 143.82
Lactate	Organic acids	223.53 ± 40.57
2-hydroxyisovalerate	Lipids	96.33 ± 23.44
Acetate	Organic acids	73.38 ± 25.19
3-hydroxybutyrate	Lipids	63.00 ± 20.58
Isoleucine	Amino acids	36.62 ± 7.19
Creatinine	Imidazolinones	30.23 ± 10.07
Leucine	Amino acids	25.63 ± 5.77
Gluconate	Organic acids	21.63 ± 11.66
sn-glycero-3-phosphocholine	Others	21.22 ± 5.58
trans-4-hydroxy-L-proline	Carboxylic acids	15.98 ± 4.73
Carnitine	Lipids	13.20 ± 10.06
3-hydroxyisovalerate	Carboxylic acids	8.63 ± 8.41
Creatine phosphate	Carboxylic acids	8.03 ± 5.17
Glycylproline	Carboxylic acids	7.96 ± 3.84
Acetoacetate	Carbohydrates	7.58 ± 4.70
Lactulose	Carbohydrates	7.03 ± 4.81
Ascorbate	Others	5.98 ± 1.58
Malonate	Carboxylic acids	5.85 ± 1.56
Creatine	Amino acids	5.70 ± 1.17
Valine	Amino acids	5.17 ± 0.21
3-methylhistidine	Others	4.30 ± 1.71
Glycolate	Lipids	4.32 ± 2.91
Levulinate	Others	3.90 ± 0.59
Acetoin	Others	3.72 ± 2.17
Succinylacetone	Organic acids	3.58 ± 1.31
2-hydroxyphenylacetate	Others	3.30 ± 1.71
5-aminolevulinate	Carboxylic acids	2.70 ± 1.50
*N*-acetylglucosamine	Carbohydrates	2.47 ± 0.06
*N*-nitrosodimethylamine	Organic acids	2.20 ± 1.67

**Table 3:** Top 30 concentration (mean ± standard deviation) of metabolites quantified (*n* ≥ 3) in urine by ^1^H-NMR spectroscopy of Hanwoo steers.

Metabolites	Class	Concentration (μM/L)
Urea	Aliphatic acylic compounds	51,262.08 ± 28,840.87
Hippurate	Amino acids	8332.20 ± 7592.61
*N*-phenylacetylglycine	Amino acids	5273.43 ± 2722.36
Glycolate	Lipids	1721.83 ± 2935.46
Trimethylamine *N*-oxide	Aliphatic acylic compounds	938.30 ± 811.19
Allantoin	Imidazolinones	769.23 ± 1019.92
2-hydroxyvalerate	Lipids	509.77 ± 354.91
Ribose	Carbohydrates	442.70 ± 312.08
Benzoate	Organic acids	427.80 ± 86.02
Glycine	Amino acids	402.90 ± 153.29
Acetate	Organic acids	310.50 ± 161.75
Guanidoacetate	Carboxylic acids	258.45 ± 239.55
Creatine	Amino acids	257.65 ± 374.33
Glucuronate	Carbohydrates	255.87 ± 178.89
Galactarate	Others	178.25 ± 119.45
Xanthine	Nucleosides, nucleotides	175.48 ± 101.52
Dimethylamine	Amines	169.97 ± 151.11
Formate	Organic acids	153.53 ± 64.46
3-indoxylsulfate	Indoles	133.60 ± 76.22
Xylitol	Carbohydrates	121.90 ± 71.35
2-methylglutarate	Lipids	105.37 ± 49.52
cis-aconitate	Carboxylic acids	102.73 ± 85.26
Glycylproline	Carboxylic acids	97.35 ± 58.11
2-hydroxyisocaproate	Lipids	94.97 ± 41.50
Mandelate	Benzoic acids	93.70 ± 31.86
Kynurenine	Amines	82.60 ± 57.39
Gentisate	Benzoic acids	78.60 ± 18.26
Phenylacetate	Organic acids	76.63 ± 30.07
Salicylurate	Benzoic acids	74.90 ± 81.77
3-phenylpropionate	Others	70.97 ± 46.43

**Table 4:** Concentration (mean ± standard deviation) of common metabolites quantified (*n* ≥ 4) in three biofluids by ^1^H-NMR spectroscopy of Hanwoo steers.

Metabolites ^a^	Class ^b^	Rumen Fluid (μM/L)	Serum (μM/L)	Urine (μM/L)
2-HPA	Others	17.55 ± 10.11	3.30 ± 1.71	31.08 ± 16.22
3-HIV	COOH	26.68 ± 22.45	8.63 ± 8.41	22.15 ± 17.84
VMA	BZA	1.37 ± 0.12	1.06 ± 0.22	19.75 ± 12.83
4-Pyridoxate	Others	6.56 ± 5.52	0.85 ± 0.19	11.98 ± 9.00
5-HIAA	Indoles	11.10 ± 6.37	2.37 ± 0.90	36.27 ± 21.16
Acetate	OA	28,172.77 ± 4924.54	73.38 ± 25.19	310.50 ± 161.75
Acetoacetate	CHO	10.85 ± 7.03	7.58 ± 4.70	60.75 ± 56.84
Anserine	AA	24.65 ± 10.43	2.18 ± 1.52	20.50 ± 5.64
Betaine	Others	1.37 ± 0.75	0.52 ± 0.30	60.43 ± 33.89
Carnitine	Lipids	14.40 ± 14.20	13.20 ± 10.06	21.13 ± 20.32
Glycylproline	COOH	45.17 ± 23.34	7.96 ± 3.84	97.35 ± 58.11
Guanidoacetate	COOH	25.90 ± 12.47	2.15 ± 2.14	258.45 ± 239.55
Isoleucine	AA	57.47 ± 22.42	36.62 ± 7.19	14.07 ± 2.72
Malonate	COOH	15.78 ± 7.15	5.85 ± 1.56	46.70 ± 44.64
NDMA	OA	12.17 ± 5.40	2.20 ± 1.67	18.95 ± 4.27
Pantothenate	COOH	7.93 ± 3.07	1.20 ± 0.26	29.35 ± 9.43
Succinylacetone	OA	8.30 ± 5.41	3.58 ± 1.31	42.15 ± 26.69
Syringate	BZA	2.63 ± 0.29	0.38 ± 0.08	11.15 ± 19.38
Thymol	Lipids	14.33 ± 4.86	1.97 ± 0.32	27.63 ± 13.40

^a^ Metabolites abbreviations: 2-HPA, 2-hydroxyphenylacetate; 3-HIV, 3-hydroxyisovalerate; VMA, 4-hydroxy-3-methoxymandelate; 5-HIAA, 5-hydroxyindole-3-acetate; NDMA, *N*-nitrosodimethylamine. ^b^ Class abbreviations: COOH, carboxylic acids; BZA, benzoic acids; OA, organic acids; CHO, carbohydrates; AA, amino acids.

## Supplementary Materials

The following are available online at https://www.mdpi.com/2218-1989/10/8/333/s1, Figure S1: The classification of identified metabolites according to chemical class rumen fluid (a), serum (b), and urine (c). Each square box color indicates the classification of metabolites; the numbers and concentration in parentheses indicated measured metabolites and sum of the total concentrations of the measured metabolites, Table S1–S4: Identified metabolites concentrations of Hanwoo steers in rumen fluid, serum, and urine samples by ^1^H-NMR spectroscopy analysis (means ± standard deviation, *n* ≥ 2), Figure S2: Principal components analysis (PCA) scores plot based on metabolites data in rumen fluid, serum, and urine by ^1^H-NMR spectroscopy analysis. On the score plot, each point represents an individual sample, with the red dot representing the rumen fluid group (*n* = 6), green dot representing the serum group (*n* = 6), and blue dot representing urine group (*n* = 4). The abscissa and ordinate represent the variance associated with PC1 and 2, respectively, Figure S3: Partial least square discriminant analysis (PLS-DA) score plot of rumen fluid, serum, and urine by ^1^H-NMR spectroscopy analysis. The shaded ellipses represent the 95% confidence interval estimated from the score. On the score plot, each point represents an individual sample, with the red dot representing the rumen fluid group (*n* = 6), green dot representing the serum group (*n* = 6), and blue dot representing urine group (*n* = 4). The abscissa and ordinate represent the variance associated with component 1 and 2, respectively.
