# Supplementary material for: Metabolomics Comparison of Hanwoo (Bos taurus coreanae) Biofluids Using Proton Nuclear Magnetic Resonance Spectroscopy
Source: Metabolites. 2020 Aug 14;10(8):333. doi: 10.3390/metabo10080333 (PMC7465992; doi:10.3390/metabo10080333)
Supplement: Supplementary file 1 [file metabolites-10-00333-s001.pdf]

Supplementary files

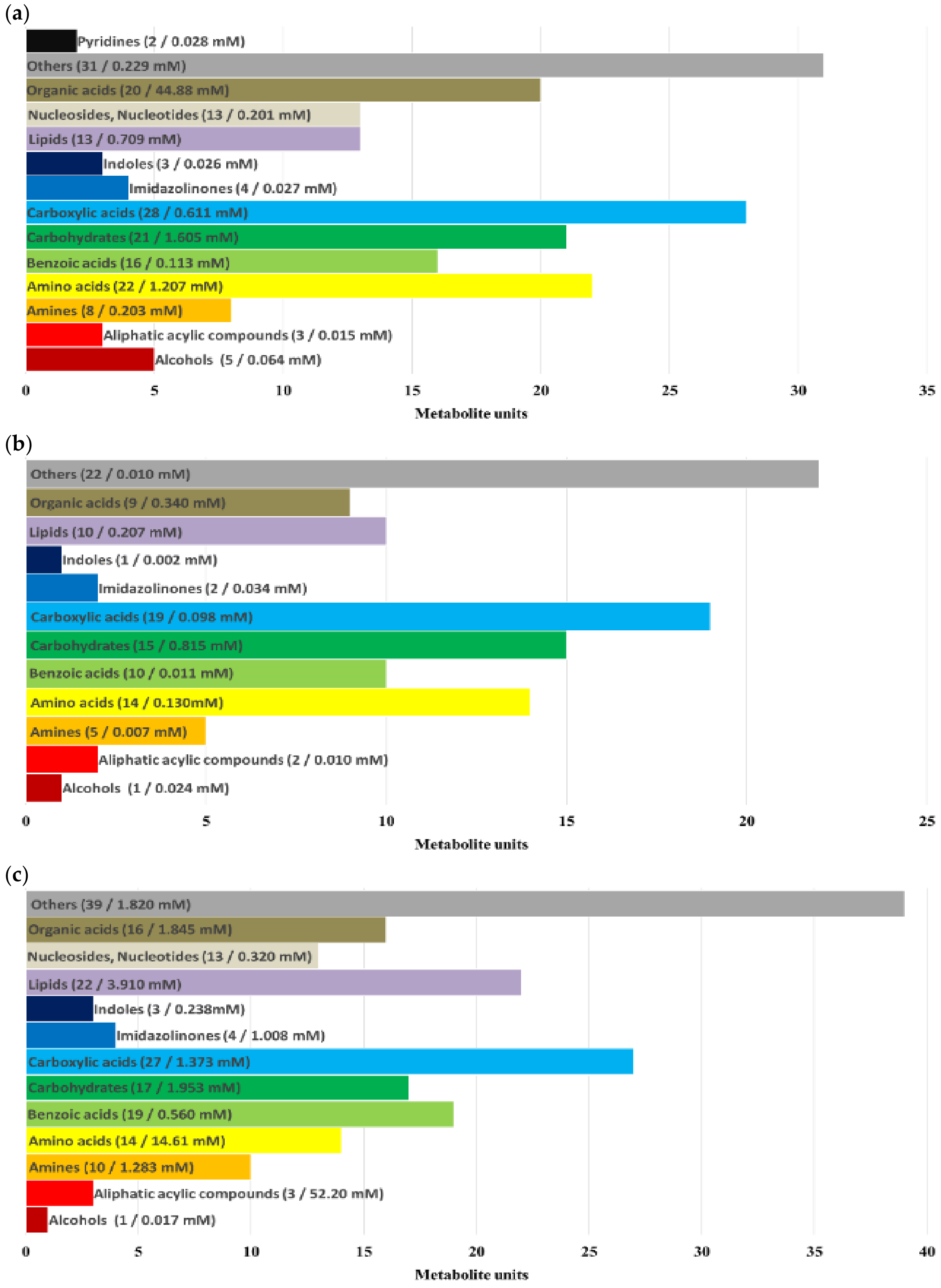

Figure S1. The classification of identified metabolites according to chemical class rumen fluid (a), serum (b), and urine (c). Each square box color indicates the classification of metabolites; the numbers and concentration in parentheses indicated measured metabolites and sum of the total concentrations of the measured metabolites.

TableS1. Identified metabolites concentration of Hanwoo steers in rumen fluid, serum, and urine samples by ^{1}H-NMR spectroscopy analysis (means ± standard deviation, $n \geq 2$)

Metabolites	Rumen fluid (μM/L)	Serum (μM/L)	Urine (μM/L)
Alcohols			
Chlorogenate	5.03 ± 1.50	Not detected (ND)	ND
Isopropanol	10.88 ± 4.13		
Aliphatic acylic compounds			
O-phosphocholine	13.00 ± 11.33	ND	3.58 ± 1.78
Trimethylamine *N*-oxide	5.27 ± 8.95		938.30 ± 811.19
Urea	86.56 ± 55.54		51262.08 ± 28840.87
Amines			
Dimethylamine	4.10 ± 3.94	ND	169.97 ± 151.11
Histamine	ND		64.23 ± 32.27
Kynurenine	17.67 ± 12.57		82.60 ± 57.39
Methylamine	42.77 ± 89.65		ND
Trimethylamine	22.02 ± 10.31		ND
Sarcosine	ND		17.45 ± 4.52
Amino acids			
2-furoylglycine	2.63 ± 0.55	ND	ND
Anserine	24.65 ± 10.43	2.18 ± 1.52	20.50 ± 5.64
Creatine	ND	5.70 ± 1.17	257.65 ± 374.33
Glycerate	52.57 ± 42.20	ND	ND
Histidine	ND	ND	37.83 ± 42.81
N-phenylacetylglycine	13.08 ± 5.08	ND	5273.43 ± 2722.36
Threonine	57.50 ± 12.14	ND	ND
1-methylhistidine	ND	4.33 ± 2.97	17.98 ± 11.36
Alanine	195.65 ± 51.88	ND	67.80 ± 30.73
Glycine	61.26 ± 41.85	ND	402.90 ± 153.29
Hippurate	ND	ND	8332.20 ± 7592.61
Isoleucine	57.47 ± 22.42	36.62 ± 7.19	14.07 ± 2.72
Leucine	36.62 ± 11.69	25.63 ± 5.77	ND
Methionine	33.54 ± 17.56	0.83 ± 0.40	ND
N-acetylglutamate	12.43 ± 8.11	ND	ND
Phenylalanine	40.87 ± 17.34	ND	ND
Proline	119.20 ± 40.69	ND	ND
Tyrosine	27.08 ± 8.59	ND	ND
Valine	41.50 ± 24.80	5.17 ± 0.21	ND
Xanthurenate	ND	ND	18.33 ± 10.09
Benzoic acids			
3,4-dihydroxybenzeneacetate	ND	ND	23.93 ± 12.43
3,4-dihydroxymandelate	ND	ND	14.00 ± 12.42
4-hydroxy-3-methoxymandelate	1.37 ± 0.12	1.06 ± 0.22	19.75 ± 12.83
4-hydroxyphenylacetate	ND	ND	60.90 ± 27.68
5-methoxysalicylate	ND	ND	3.07 ± 2.93
Acetylsalicylate	14.56 ± 6.56	ND	21.13 ± 31.41
Gentisate	ND	ND	78.60 ± 18.26
Homogentisate	ND	ND	11.20 ± 4.16
Mandelate	2.18 ± 0.29	ND	93.70 ± 31.86
o-Cresol	ND	ND	18.70 ± 10.66
p-Cresol	12.50 ± 5.38	ND	34.85 ± 7.60
Salicylurate	ND	ND	74.90 ± 81.77
Syringate	2.63 ± 0.29	0.38 ± 0.08	11.15 ± 19.38
Tartrate	ND	1.13 ± 0.21	5.33 ± 1.70
Vanillate	ND	1.03 ± 0.15	11.67 ± 13.73

Table S2. *Cont.*

Metabolites	Rumen fluid (μM/L)	Serum (μM/L)	Urine (μM/L)
Carbohydrates			
1,3-dihydroxyacetone	16.62 ± 7.51	ND	ND
Acetoacetate	10.85 ± 7.03	7.58 ± 4.70	60.75 ± 56.84
Erythritol	29.98 ± 3.48	ND	ND
Fructose	28.00 ± 4.49	ND	ND
Galactitol	29.52 ± 10.94	ND	ND
Galactose	34.00 ± 33.73	ND	ND
Glucose	632.42 ± 387.16	603.60 ± 143.82	ND
Glucuronate	ND	ND	255.87 ± 178.89
Lactulose	52.36 ± 40.70	7.03 ± 4.81	ND
Maltose	178.13 ± 278.73	ND	ND
Mannose	17.30 ± 13.66	ND	ND
N-acetylglucosamine	112.55 ± 16.91	2.47 ± 0.06	ND
Pyruvate	51.98 ± 27.61	ND	ND
Ribose	231.48 ± 50.74	ND	442.70 ± 312.08
Succinate	22.75 ± 8.82	ND	9.03 ± 10.10
Sucrose	ND	ND	54.65 ± 28.08
Xylitol	ND	ND	121.90 ± 71.35
Xylose	ND	ND	44.17 ± 8.15
Carboxylic acids			
2-hydroxyisobutyrate	7.72 ± 3.66	ND	10.57 ± 7.06
3-hydroxyisovalerate	26.68 ± 22.45	8.63 ± 8.41	22.15 ± 17.84
3-hydroxyphenylacetate	15.15 ± 6.60	ND	42.10 ± 16.40
3-methyl-2-oxovalerate	2.10 ± 0.35	ND	ND
5-aminolevulinate	ND	2.70 ± 1.50	12.15 ± 6.58
Alloisoleucine	38.13 ± 11.27	ND	ND
cis-aconitate	ND	ND	102.73 ± 85.26
Creatine phosphate	ND	8.03 ± 5.17	65.98 ± 17.87
Glycylproline	45.17 ± 23.34	7.96 ± 3.84	97.35 ± 58.11
Guanidoacetate	25.90 ± 12.47	2.15 ± 2.14	258.45 ± 239.55
Homovanillate	5.66 ± 2.55	ND	32.20 ± 20.58
Hydroxyacetone	ND	0.83 ± 0.19	9.67 ± 5.98
Malonate	15.78 ± 7.15	5.85 ± 1.56	46.70 ± 44.64
N,N-dimethylglycine	ND	ND	11.53 ± 16.43
*N*6-acetyllysine	30.18 ± 29.98	ND	ND
N-acetylaspartate	9.18 ± 6.81	ND	ND
N-acetylcysteine	24.55 ± 1.65	ND	ND
N-acetylglycine	61.42 ± 64.75	ND	ND
N-acetylornithine	41.63 ± 32.67	ND	ND
N-acetyltyrosine	ND	ND	22.13 ± 24.64
N-carbamoylaspartate	86.07 ± 33.83	ND	ND
N-phenylacetylphenylalanine	8.34 ± 1.32	ND	ND
Pantothenate	7.93 ± 3.07	1.20 ± 0.26	29.35 ± 9.43
trans-aconitate	6.87 ± 1.79	ND	18.97 ±14.16
trans-4-hydroxy-L-proline	ND	15.98 ± 4.73	ND
N-alpha-acetyllysine	51.08 ± 53.04	ND	ND
Imidazolinones			
Allantoin	ND	ND	769.23 ± 1019.92
Creatinine	ND	30.23 ± 10.07	34.57 ± 21.78
Imidazole	5.84 ± 3.88	ND	46.03 ± 11.57
Indoles			
3-indoxylsulfate	6.17 ± 3.93	ND	133.60 ± 76.22
5-hydroxyindole-3-acetate	11.10 ± 6.37	2.37 ± 0.90	36.27 ± 21.16

Table S3. *Cont.*

Metabolites	Rumen fluid (μM/L)	Serum (μM/L)	Urine (μM/L)
Lipids			
2-hydroxyisocaproate	ND	ND	94.97 ± 41.50
2-hydroxyisovalerate	ND	96.33 ± 23.44	ND
2-hydroxyvalerate	ND	ND	509.77 ± 354.91
2-methylglutarate	ND	ND	105.37 ± 49.52
3-hydroxybutyrate	ND	63.00 ± 20.58	ND
3,5-dibromotyrosine	42.88 ± 26.62	ND	ND
3-hydroxy-3-methylglutarate	51.50 ± 33.29	ND	34.10 ± 23.40
3-methylglutarate	214.75 ± 67.05	ND	ND
Carnitine	14.40 ± 14.20	13.20 ± 10.06	21.13 ± 20.32
Caprate	160.80 ± 58.72	ND	ND
Choline	3.10 ± 1.67	ND	9.90 ± 3.68
Ethylene glycol	13.58 ± 11.45	ND	23.73 ± 32.81
Glutaric acid monomethyl ester	ND	ND	15.33 ± 22.18
Glycolate	ND	4.32 ± 2.91	1721.83 ± 2935.46
Methylsuccinate	ND	ND	45.85 ± 18.75
O-acetylcarnitine	16.03 ± 15.55	ND	10.27 ± 7.98
Thymol	14.33 ± 4.86	1.97 ± 0.32	27.63 ± 13.40
Nucleosides, Nucleotides			
Hypoxanthine	25.80 ± 11.72		ND
Uracil	58.87 ± 17.19	ND	ND
Xanthine	70.03 ± 12.87		175.48 ± 101.52
Organic acids			
3-hydroxykynurenine	15.83 ± 5.83	ND	ND
Acetamide	237.18 ± 47.79	ND	ND
Acetate	28172.77 ± 4924.54	73.38 ± 25.19	310.50 ± 161.75
Benzoate	12.43 ± 0.15	ND	427.80 ± 86.02
Butyrate	6021.97 ± 1140.22	ND	ND
Ferulate	3.22 ± 2.25	ND	ND
Formate	ND	ND	153.53 ± 64.46
Gluconate	43.90 ± 21.04	21.63 ± 11.66	ND
Isobutyrate	495.55 ± 168.30	ND	ND
Isovalerate	470.08 ± 144.26	ND	ND
Lactate	ND	223.53 ± 40.57	ND
Nicotinate	20.06 ± 4.71	ND	ND
Nicotinurate	5.07 ± 1.96	ND	ND
N-nitrosodimethylamine	12.17 ± 5.40	2.20 ± 1.67	18.95 ± 4.27
O-acetylcholine	ND	0.55 ± 0.37	2.38 ± 1.21
Phenylacetate	220.22 ± 49.20	ND	76.63 ± 30.07
Propionate	8126.70 ± 1341.28	ND	ND
Succinylacetone	8.30 ± 5.41	3.58 ± 1.31	42.15 ± 26.69
Valerate	940.82 ± 187.60	ND	ND

Table S4. *Cont.*

Metabolites	Rumen fluid (μM/L)	Serum (μM/L)	Urine (μM/L)
Others			
1,3-dimethylurate	2.93 ± 1.85	ND	3.75 ± 1.91
1,7-dimethylxanthine	ND	1.03 ± 0.26	ND
2-hydroxyphenylacetate	17.55 ± 10.11	3.30 ± 1.71	31.08 ± 16.22
2-oxocaproate	4.50 ± 1.04	ND	ND
3-methylxanthine	ND	ND	4.63 ± 5.95
3-phenylpropionate	223.40 ± 55.69	ND	70.97 ± 46.43
4-pyridoxate	6.56 ± 5.52	0.85 ± 0.19	11.98 ± 9.00
Acetoin	ND	3.72 ± 2.17	ND
Acetone	ND	0.80 ± 0.80	ND
Ascorbate	ND	5.98 ± 1.58	ND
Betaine	1.37 ± 0.75	0.52 ± 0.30	60.43 ± 33.89
Biotin	45.33 ± 9.28	ND	ND
Butanone	ND	ND	18.70 ± 6.00
Caffeine	7.98 ± 2.76	ND	6.80 ± 1.50
Cellobiose	12.15 ± 13.74	ND	ND
Dimethyl sulfone	1.78 ± 1.63	ND	24.10 ± 16.16
Epicatechin	ND	ND	5.37 ± 3.59
Galactarate	ND	ND	178.25 ± 119.45
IMP	ND	ND	6.90 ± 3.84
Indole-3-acetate	8.53 ± 3.87	ND	56.77 ± 39.19
Indole-3-lactate	9.10 ± 2.88	ND	54.23 ± 35.99
Levulinate	ND	3.90 ± 0.59	ND
Melatonin	8.04 ± 0.66	ND	ND
N-acetylserotonin	ND	1.27 ± 0.38	23.53 ± 8.35
N-methylhydantoin	ND	0.93 ± 0.15	6.30 ± 6.68
Pyridoxine	ND	ND	7.10 ± 1.67
Riboflavin	ND	ND	8.55 ± 4.81
Theophylline	1.30 ± 0.50	ND	ND
sn-glycero-3-phosphocholine	ND	21.22 ± 5.58	13.40 ± 12.08
3-methylhistidine	ND	4.30 ± 1.71	70.25 ± 68.37

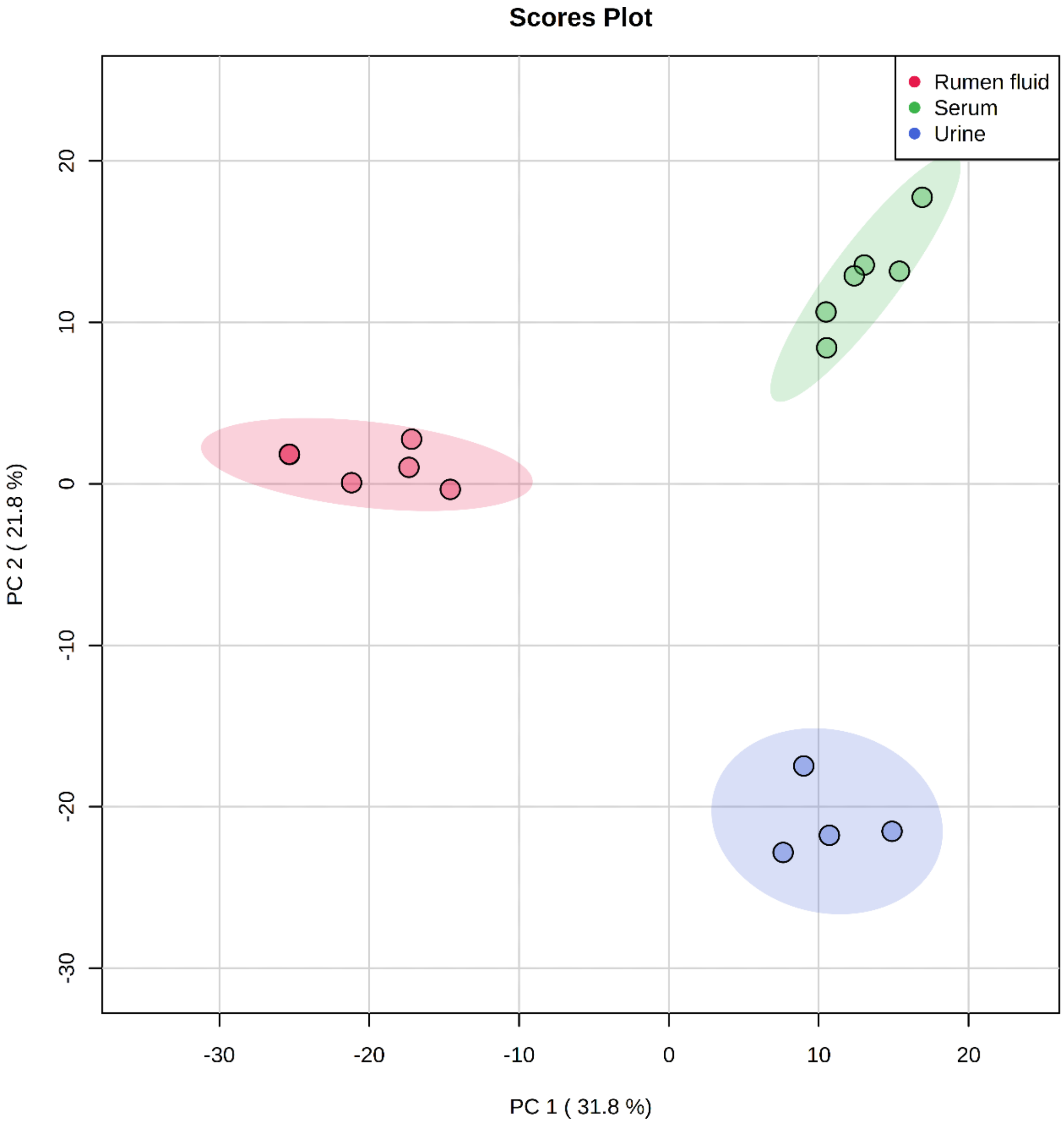

Figure S2. Principal components analysis (PCA) scores plot based on metabolites data in rumen fluid, serum and urine by ^{1}H-NMR spectroscopy analysis. On the score plot, each point represents an individual sample, with the red dot representing the rumen fluid group (n = 6), green dot representing the serum group (n = 6), and blue dot representing urine group (n = 4). The abscissa and ordinate represent the variance associated with PC 1 and 2, respectively

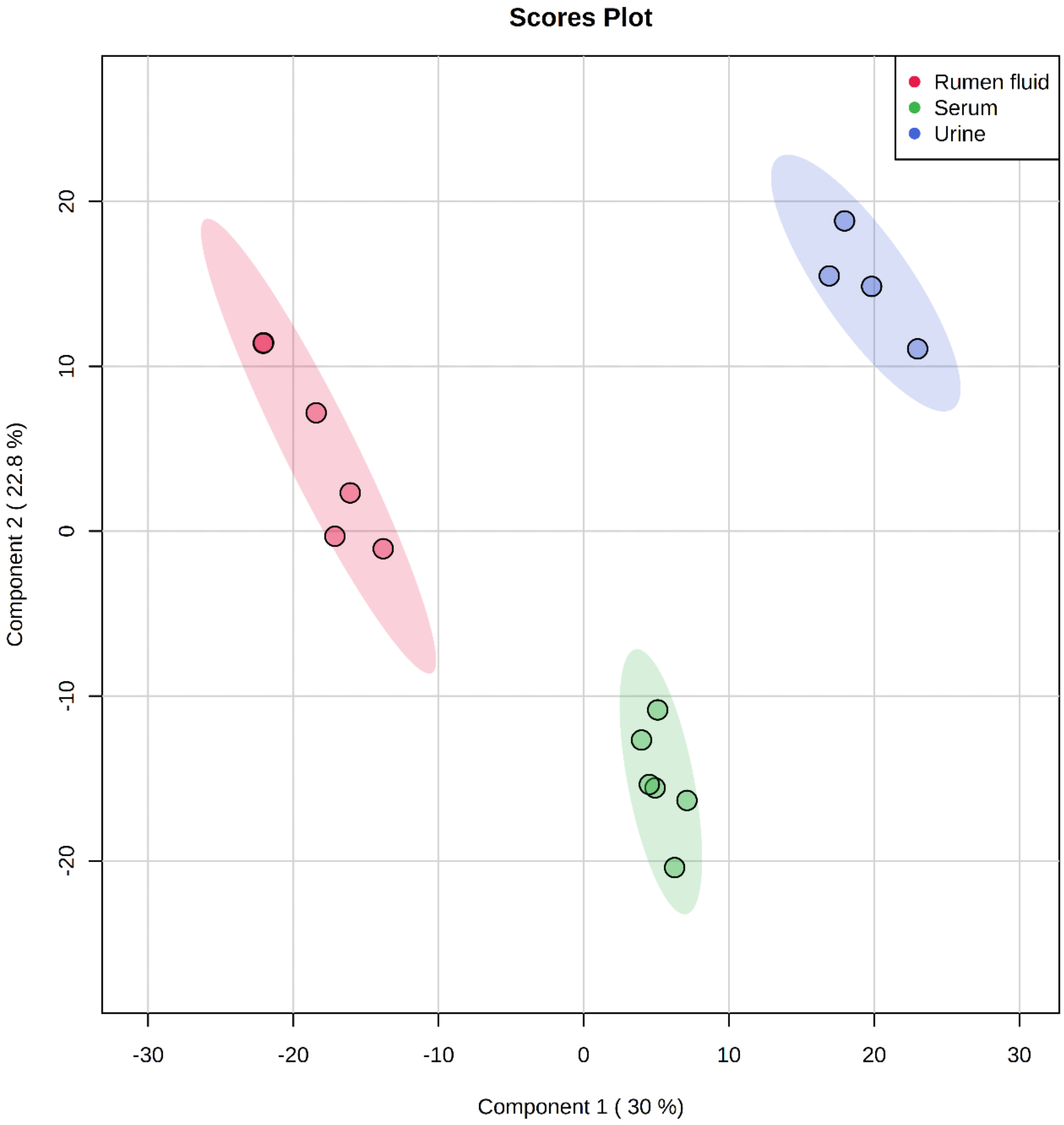

Figure S3. Partial least square discriminant analysis (PLS-DA) score plot of rumen fluid, serum, and urine by ^{1}H-NMR spectroscopy analysis. The shaded ellipses represent the 95% confidence interval estimated from the score. On the score plot, each point represents an individual sample, with the red dot representing the rumen fluid group (n = 6), green dot representing the serum group (n = 6), and blue dot representing urine group (n = 4). The abscissa and ordinate represent the variance associated with components 1 and 2, respectively.
